# Shaping Healthy Eating Habits in Children With Persuasive Strategies: Toward a Typology

**DOI:** 10.3389/fpubh.2021.676127

**Published:** 2021-09-08

**Authors:** Alice Binder, Brigitte Naderer, Jörg Matthes

**Affiliations:** ^1^Department of Communication, Faculty of Social Sciences, University of Vienna, Vienna, Austria; ^2^Department of Media and Communication, Ludwig Maximilian University of Munich, Munich, Germany

**Keywords:** food advertising, healthy food behavior, message factors, persuasive strategies, children, typology

## Abstract

There is an abundance of evidence that the presentation of unhealthy foods (UHFs) in different media has the power to shape eating habits in children. Compared to this rich body of work with regard to the effects of UHF presentations, studies testing the effects of healthy foods (HFs) are less conclusive. In particular, while the persuasive mechanisms behind HF presentations are well-understood, we lack insights about the role of messages factors, that is, how are (and should) HFs (be) presented in order to foster healthy eating habits in children. This paper tackles this research gap by suggesting the Persuasive Strategies Presenting Healthy Foods to Children (PSPHF) typology, classified along three pillars: (a) composition-related characteristics, (b) source-related characteristics, and (c) information-related characteristics. Against the background of the PSPHF typology, we review the available empirical evidence, outline pressing research gaps, and discuss implications for researchers, health promoters, and program planers.

Food is an essential part of our life, and our food environment shapes preferences and eating behaviors. The social environment and cultural environment are essential factors in shaping the eating behaviors of children (1). Yet, food preferences can also be formed in a mediated environment (2). The depiction of foods in the media has been heavily criticized in the past (3). Content analyses of traditional TV commercials (3, 4), of online content (5), of embedded forms of advertising (6), and food depictions within entertaining content like TV series (7), or movies (8), indicate a dominant focus on food low in nutritional value and high in fat, salt, and/or sugar. Keller and Schulz (9) thus ascertain that the media presents a distorted view of the types and proportions of foods that should be eaten.

The lack of foods high in nutritional value (e.g., fruits and vegetables) and the simultaneously high focus on fast food, candy, soft drink, alcohol, and salted snacks in mass media are a cause for concern. Particularly, children are not consuming the recommended fruit and vegetable ratios, but tend to overeat sugary and salty products (10). The predominant unhealthy food (UHF) depictions in the media might reinforce the already-existing eating preferences of children. This is indicated by a recent meta-analysis (11) and two literature reviews (12, 13) demonstrating that the eating habits of children are heavily affected by food presentations in the media. In the long run, this can negatively affect the weight of the children, increasing the likelihood of obesity and overweight, which raises serious implications for long-term health concerns (14).

Existing research has shown that children respond to the presentation of unhealthy snacks in their corresponding food behaviors and preferences (15–18). Fostering healthy food (HF) behaviors through promotional efforts has proven to be less effective and not as straightforward. Presentations of HF in media content targeted at children, while able to increase the hypothetical liking of these foods (19, 20), however, have limited or even backfiring effects on food choices (17, 21). Researchers have argued that the mere presentation of HFs is not sufficient to shape eating habits among children. By contrast, food presentations need to be connected to specific persuasive strategies (22).

A systematic analysis of such persuasive strategies is highly warranted. We have a good grasp of the underlying mechanisms and of the individual susceptibility factors explaining the effects of food presentations on children (23, 24). However, despite these efforts, and the general literature on message factors in health communication (25, 26), we lack a comprehensive overview of the message factors that can be applied to HF-related media content targeted at children. Research on message factors from other areas such as non-food products or research on adults cannot be generalized to the presentation of HFs to children. In this paper, we therefore suggest a comprehensive typology of persuasive strategies, the Persuasive Strategies Presenting Healthy Foods to Children (PSPHF) typology. We present an integration of the available empirical findings into our typology and discuss how different persuasive strategies in connection with HF presentations can shape attitudinal, intentional, and behavioral outcomes in children. The PSPHF typology has not only important theoretical and methodological implications for future research, but also potentially informative to practitioners and policy regulators. That is, in contrast to individual (e.g., age) and contextual (e.g., parents, culture) factors that also shape healthy eating habits of children, persuasive strategies can be strategically implemented by content creators.

## Theoretical Background

To gain insights into the underlying mechanisms of food presentations, Folkvord and colleagues (24) developed a theoretical framework focusing on the effectiveness of promotional strategies. Based on the Cue Reactivity Theory (27), and on the Processing of Commercialized Media Content model (PCMC) (28), the Reactivity of Embedded Food Cues in Advertising Model (REFCAM) (24) suggests that specific foods integrated into editorial content, such as product placements or food integrated into advergames, influence children in a two-step process. In a first step, physiological (i.e., heart rate) (29) and/or psychological reactions (i.e., thought about foods) (30) of the children are influenced by the presentation of foods. Focusing on embedded foods, the model assumes that decreased cognitive processing, thus enhanced automatic processing, can influence the eating behavior of children in a next step (24). The authors describe the relationship between children's reactions toward the presented foods (i.e., cue reactivity) and children's eating behavior as being reciprocal, thus, as an “incentive-sensitization process” (p. 27) (24). Moreover, based on the Differential Susceptibility to Media Effects Model (31), the authors assume that individual susceptibility factors, such as children's Body Mass Index (BMI), are influencing this two-step process. Very briefly, the authors mention that message factors, i.e., “the level of integration of food cues,” plays a role in children's reactions as these factors are influencing “the level of elaboration” (p. 28) (24). However, this is not discussed in more detail in the REFCAM.

Focusing on the effectiveness of healthy food presentations in media, Folkvord (23) established another theoretical framework. Similar to the REFCAM (24), the Promotion of Healthy Foods Model describes a two-step process: In a first step, the attention as well as the “reinforced value of the [HF] (i.e., liking and wanting)” has to be influenced before the food presentations contribute to a healthy eating behavior (p. 114). Again, the author suggests a reciprocal relationship between the reinforced value as well as the food intake. Individual susceptibility factors (e.g., BMI) as well as contextual factors (e.g., parental background) (23) of the children are again considered in the model.

In light of the existing theoretical conceptualizations (23, 24) as well as the available empirical evidence (17, 18, 30, 32), we have a good understanding of *the underlying processes* behind the effectiveness of food presentations in the media. However, when it comes to HF presentations, we particularly lack insights into how variations in content shape the openness of the children to HF options. That is, we need a typology that can be used to describe how media messages regarding HFs *should* be arranged to successfully increase the attractiveness of these foods for children. More precisely, we lack an in-depth understanding of most important *message factors*, thus, the most effective “level of integration of HF cues” (p. 28) (24).

## A Typology of Message Factors

Based on content analyses that investigated the presentation of foods (4, 6, 8, 9, 33), and based on current literature reviews regarding persuasive techniques used in food promotions with children (34), we have identified three pillars of persuasive strategies as our PSPHF typology: (a) *composition-related characteristics*, (b) *source-related characteristics*, and (c) *information-related characteristics*.

**Figure 1:**
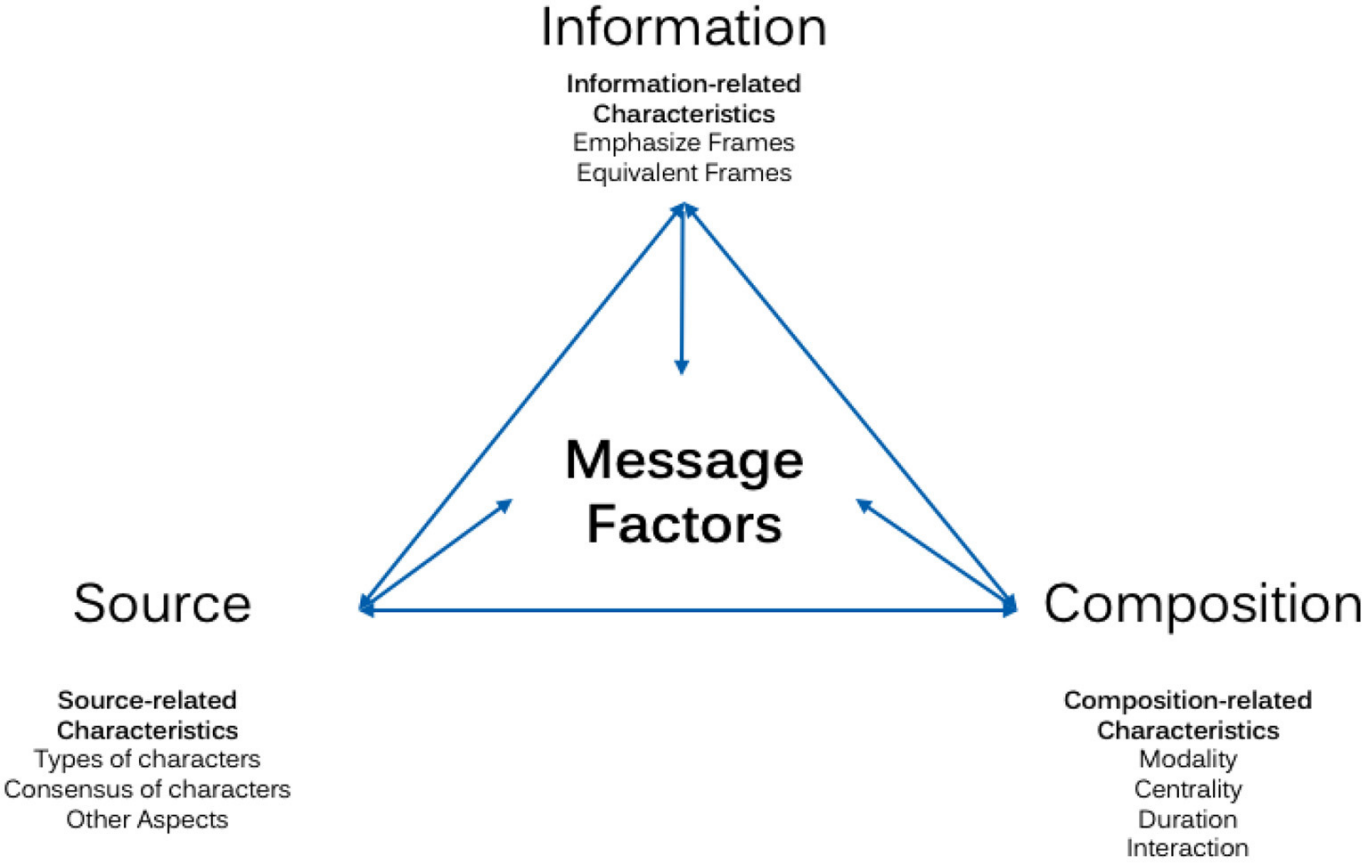
Typology of persuasive strategies.

*Composition-related characteristics* are composed of the modality of (visual, audio, audiovisual) (35), the centrality of (foreground, background) (36), duration of (36), and interaction with (37) foods. This pillar mainly focuses on theoretical and empirical assumptions of obtrusiveness and awareness of HF presentations as motivational factors to contribute to healthier eating. Closely connected to the effectiveness of interactive elements, *source-related characteristics* include strategies that are directly related to the source who is presenting the message. This second pillar describes who is providing the message (38), but also other source-related aspects, such as how many characters/endorsers are presented in connection with foods (39). While the majority of effects might depend again partly on the awareness and obtrusiveness as motivational factors, the effectiveness of who is presented in connection with an HF depends on the relationship of children with the presenter (40). Emotional factors can be described as the main drivers of the effectiveness of these strategies. Lastly, the *information-related characteristics*, i.e., which information is connected with a HF presentation, include aspects of which arguments are presented to portray the importance of consuming a specific food, and also how the information is presented (41). Depending on the strategic integration of the information, cognitive but also emotional components act as motivational factors. The message factors are intertwined; thus, a combination is possible, and effects may not be independent (see Figure 1).

The three dimensions proposed capture important possibilities of how HFs can be integrated in the media in order to influence healthy eating habits of children. These components are especially important as soon as children are able to understand the content (i.e., have the ability to process the provided information) and to build relationships with, e.g., the characters presented in this content. Based on the current empirical evidence, this developing process begins at the age of three and is seen as completed at the approximate age of 16 when adolescents have reached the end of the so-called reflective phase (42). Of course, the following strategies might differ in their effectiveness depending on the developmental stages of children. However, this paper does not specifically predict for which age group-specific HF presentations are effective. The paper rather aims to provide an overview of possible effective strategies based on the available empirical evidence and current theoretical assumptions.

## Literature Review

### Composition

Research on embedded brands highlights the relevance of composition factors for the effectiveness of cue integrations in entertaining content. In particular, four aspects have been identified: (1) the modality (35), (2) the centrality (36), (3) the duration of a presentation (35), and (4) interactive elements of the presentation (43).

#### Modality

Three presentation modalities are typically distinguished: (a) visual, (b) verbal, or (c) audiovisual presentations (35). The order of these modality types already reflects their rising obtrusiveness. Recall ability of information and conscious awareness are considered as relevant when making product decisions (35, 44). Thus, particularly, audiovisual presentations are considered as effective with regard to awareness and memory measures due to the double modality of the presentation (35). Of course, there is also an argument to be made about the effectiveness of unobtrusive presentations. This is founded on the mere exposure effect, which is based on the theoretical concept of a non-associative learning process (45). The mere exposure effect shows a positive affective effect due to multiple, unobtrusive stimulus presentations on the stimulus evaluation without explicit memory traces (46).

Yet, conscious awareness for HF might be particularly relevant (47). Especially when being asked to choose between a HF and an UHF option, conscious awareness for the former might be crucial, as children have to act against their inherent preference to choose the latter (48). Children have to activate their inhibitory control and consciously remind themselves of the healthier option. Along these lines, Charry (19) has indicated that multimodal, i.e., audiovisual HF presentations are more effective in creating HF intentions in children compared to unimodal, i.e., visual presentations. She explains this effect due to the higher level of attention multimodal food presentations create. We thus follow Charry's (19) (p. 611) recommendation: “that screenwriters of popular programmes should be advised to use audio-visual supports, not those that are merely visual, when integrating HF consumption messages into their shows for pre-adolescents.” It should be noted that this study (19) was focusing on the intentional and not actual behavior of the children. Thus, a systematic analysis of how modality drives HF choices in children is a gap in research that should be addressed in the future.

#### Centrality

To evaluate how central a cue is presented, research often distinguishes whether or not the cue is presented (a) in the background (i.e., second image plane), (b) as a central presentation (i.e., first image plane), and (c) as a closeup (i.e., depiction on the first image plane and on more than 50% of the screen) (6).

Based on the theory of selective attention, “we perceive and remember only those objects and details that receive focused attention” (p. 1059) (49). Presentation centrality might be relevant for young children, as they have only a limited attention span (28) and thus might focus particularly on foods in the foreground. With regard to HF presentations, we again stress the role of conscious awareness for HF (47), in order to assure that children are able to activate their inhibitory control and consciously remind themselves of healthier food options (48). We would thus argue for a central and prominent integration of HF instead of a mere fruit platter in the background. Studies that systematically test these assumptions for HF presentations in children are still missing.

#### Duration

How long a certain cue is shown within an entertaining context also speaks to the obtrusiveness of a presentation. For brand placements, we again see that longer brand presentations lead to higher levels of awareness and memory. This, however, also coincides with more counterarguing against the presented brand and more negative brand associations (36). Yet, for HF presentations, obtrusiveness is, as highlighted already, relevant in order to create awareness and to positively influence HF choices (47). Hence, following this line of argumentation, we would suggest that for HF presentations more is actually more and we recommend longer air-time for fruit and vegetables (8). However, again we clearly lack empirical evidence for these assumptions with children as the target group.

#### Interaction

In addition to the already-mentioned composition factors, interaction with a cue (in our case a food) is regarded as important (37). Interaction is defined as the action of a character who is using, holding, handling (e.g., preparing and requesting), or eating a product (50). In the PCMC model, the authors (28) postulate that children allocate more cognitive resources toward more interactive than non-interactive presentations.

There are three theoretical explanations for why interactive placements are particularly successful. First, interaction with food presents the utilization of the product (51). This, in turn, facilitates knowledge about the characteristics of the product. The observation of how a media character interacts with a product may then lead to a modeling behavior (52). Second, the concept of Para-social Interaction (PSI) (40) and the Balance Model (37) suggests that if media characters evaluate products, this could affect product assessments of viewers because of the connection to the presented characters (36, 37). Thus, when children build a relationship with a character, and this character interacts with a product, the young audience is likely to adopt the behavior of the character due to this identification process. Third, interactive placements can create a meaning transfer from the presented character to the product. Therefore, likable characters, which are typically found in content targeted at children, can transfer their popularity onto the product. This is based on the theory of Evaluative Conditioning (53). Conditioning occurs when a liked character repeatedly is associated with a product (53), and it has been shown to positively influence the product evaluation.

Some effect studies on both product presentations and UHF placements with adults (36, 37) and children (43) have shown that interactive and prominent presentations trigger product choices to a higher extent compared to non-interactive presentations. One recent study revealed that showing HFs in a social context led to higher memory among children. Moreover, presenting HFs in a gregarious context led to the best memory of the HFs (54). Along the same lines, Spielvogel et al. (32) revealed that interactive presentations compared to non-interactive ones are more effective in arousing visual attention for HFs. This underlines the importance of interactive integrations of HFs in content targeted at children. Still, more studies are needed to replicate these first findings.

### Source

When investigating the effects of interactive elements, the source, i.e., characters or endorsers, interacting with products seems to be a possible important motivator. As described earlier, based on the concept of PSI (40) and the Balance Model (37), characters who interact with products might influence the healthy eating habits of children. This process might be based on the liking (55), the similarity evaluation (56), the physical attractiveness (57), and also on the perceived credibility (58) of these characters. Two components seem especially important: (1) who is presented in connection with the HFs (38) and (2) how many characters are connected to the product (59).

#### Types of Characters/Endorsers

According to the Social Cognitive Theory (51), children learn and in a further step model behaviors observed in their surroundings. This modeling behavior can be also influenced *via* symbolic learning, thus, with behaviors presented by media characters (60). This behavior might vary according to the characters presented with HFs. Based on Friedman and Friedman (38) media, characters can be categorized into three important categories: peers, celebrities, and experts.

*Peers*. Especially when it comes to eating behaviors, peers have been found to be effective (60, 61). Peers can be defined “as children roughly the same age as the children themselves” (p. 42) (22). Studies argue that with increasing age parents get less important, while the influence of peers increases (62). This is argued based on the goal of affiliation (i.e., positive relationship with friends, gaining popularity) (63). Moreover, the perceived similarity between a peer and a child can act as an important driver to copy a behavior (58). In this regard, studies revealed that presenting peers in connection with HFs can act as a driver for healthy eating habits in children (22, 64). Thus, connecting HFs in media with peers might be one effective strategy. However, research should replicate these first results and study in more detail the underlying mechanism.

*Celebrities*. Celebrities are widely used when presenting foods to children (5). Studies agree that celebrities or other prominent/popular characters (65) are very attractive promotional figures. Thus, licensed characters (i.e., Ronald McDonald) (65), traditional celebrities (e.g., top-athletes) (66), or the so-called social media influencers (67) have the potential to impact the eating habits of children. The effectiveness is driven by the assumption that people tend to feel the wish to emulate the lifestyle of these popular media figures (68). Many studies already revealed the effectiveness of prominent characters for UHF (69). Testing the effects on attitudes and intentional behaviors, some studies showed positive effects of HFs in connection with popular characters (70). However, testing real behaviors or attentional effects, the connection of popular characters with HFs compared to UHFs showed mostly less effectiveness or non-significant effects (71–73). There is a big variety of celebrity endorsers (e.g., licensed figures, traditional celebrities, or social media influencers); thus, investigating these different types in connection with HFs seems essential to gain a better picture of which celebrities can be deemed as effective. Overall, connecting HFs with celebrities could be a good strategy to influence the healthy eating habits of children.

*Experts*. Another type of character that is theoretically assumed as being effective is experts (38). In the field of health communication, experts can be defined as medical doctors, or other persons working in health-related jobs (i.e., dietitian) (58). The effectiveness of experts is theoretically based on the perceived competence or knowledge (58) and the perceived authority (61). Taking nutritional advice from an unfamiliar source certainly has a larger impact if this source has relevant credentials. According to one study comparing the three types of social endorsers (peers, celebrities, and experts), experts proofed particularly effective in shaping the healthy eating habits of children (74). However, the study used only unfamiliar characters, which might have led to the ineffectiveness of celebrities and peers (15).

In sum, connecting HFs with media characters might be a good strategy to motivate children to eat HFs. Liking, familiarity, credibility, or attractiveness of the social endorsers (75) are the factors that have the ability to shape the overall effectiveness of HF presentations regardless of the type of character.

#### Consensus of Characters/Endorsers

Based on assumptions of conformity concepts (76), majority-biased transmission (39), as well as on the spiral of silence (77), the modeling of behaviors might increase when a majority of people are conducting a specific behavior. Thus, not only the type of endorser might influence healthy eating habits of children but also how many characters endorse eating HFs. There exist many assumptions why people tend to model the behavior of a majority. For example, the “copy-the-majority tactic” describes that people tend to behave according to a majority because they simply think it is the majority (78); or random copying means that “if observers copy an individual at random, the likelihood to copy a majority member exceeds that of copying a minority member” (p. 65) (39). Along the same lines, other social factors might influence the copying of the majority such as the wish for prestige (79).

Some studies revealed that presenting a majority of characters or transporting social norms of a majority in media connected with HFs can shape the healthy eating behaviors of the children (22, 61). Therefore, this might be a fruitful avenue to increase HF consumption of children with different sources.

### Information

The level of integration can not only vary based on compositional factors or source-related factors. Also which information is connected with a HF presentation can influence the reactions of the children (30, 80). Current studies can be roughly classified along with two forms of presentation: (1) what information is connected with HFs (i.e., emphasis frames) and (2) how identical chunks of information are presented (i.e., equivalence frames) (41). While emphasis frames highlight some information regarding HF while not mentioning others, equivalence frames describe the same information in different ways.

#### Emphasis Frames

There are many persuasive strategies with the goal to emphasize some aspects of a message while not mentioning others. Based on the Elaboration Likelihood Model (ELM) (81), people base product decisions either more on affects or cognitions. More precisely, while affects describe positive or negative emotions toward a product, cognitions include positive or negative attributes which are accredited to a product (82). In the research area of health communication, some empirical evidence has shown that positive affective cues are especially effective when promoting health-related behaviors (30, 83, 84), while cognitive cues fall short in comparison (30, 84) in adults as well as in children. Thus, emphasizing the taste and affective components connected to a food proves more impactful than highlighting nutritional facts (i.e., “full of vitamins”).

Another persuasive strategy is the use of threat or fear appeals. The Protection Motivation Theory (85) describes that fear or threat appeal is especially effective if (a) the threat is perceived as realistic, (b) the fulfillment of the threat is appraised as likely, (c) the presented solution for the portrayed problem is evaluated as efficient, and (d) the presented solution is assessed as realizable. A study with children showcased threat appeals connected to obesity are an effective strategy in influencing the healthy eating behavior of children (86). Thus, while ethically questionable (87), threat appeals seem to be effective in influencing the healthy eating behaviors of children.

Often health-related content also emphasizes the positive effects of a specific behavior on the appearance of an individual, or on the health of an individual (88). While some studies showed the effectiveness of such appearance frames in adults (89), this strategy has not been studied in children, leaving this as a yet unexamined line of research.

Furthermore, when advertising UHFs, positive outcomes of consumptions, such as fun, vitality, or sociability, are typically emphasized (90). These strategies do also have the potential to present HFs in persuasive ways. However, connecting HFs with positive outcomes such as fun, vitality, or sociability has not been properly investigated to date.

#### Equivalent Frames

One persuasive strategy often used when presenting health-related topics is to either present the gain of engaging in a specific behavior or the loss when not following this behavior (25, 26). According to the Prospect Theory (91), emphasizing the losses are especially effective for deductive behaviors (e.g., getting a mammography), while using gain frames are more effective for preventative behaviors (e.g., preventing obesity by eating healthy). This assumption is based on different degrees of risk assessment of these behaviors: While detection behaviors potentially involve high financial costs as well as possible negative consequences and are therefore evaluated as risky, prevention behaviors involve little financial costs and not conducting this behavior could be risky (92). Existing studies already indicate that presenting gain arguments in connection with HF presentations in media presents a good strategy to influence intentions and real consumption behavior of the children positively (80, 93). Therefore, more gain arguments should be used in HF presentations.

Another persuasive strategy based on equivalent framing is to either present a reward or a punishment for a specific behavior. This follows the classic assumptions of the Social Cognitive Theory that deduces that negative consequences make a certain behavior unappealing, while receiving rewards positively reinforces the conducted actions (2). This assumption has been tested for nutritional behavior in adults, particularly regarding alcohol portrayals in the media. Bahk (94), for instance, found that compared to showing no depictions of alcohol consumption, the presence of negative consequences deteriorated attitudes toward alcohol. Yet, the mere absence of negative consequences and the presentation of positive consequences improved the alcohol evaluations of the participants (95). A recent study furthermore indicates that either positive or negative consequences affect what behavioral expectancies viewers link to the consumption of alcohol (96). These results highlight the relevance of consequence portrayals. We are not, however, aware of any studies that employ this technique in a content setting targeted at HF and children. Still, from these first results, we conclude that presenting HFs as rewards might act as a good motivational cue for children.

For an overview of the empirical evidence of HFs presentations and its effects on children and the remaining research gaps see Tables 1, 2.

**Table 1:** Overview of evidence on the persuasive strategies regarding the effects of healthy foods on children.

		**Outcomes**		
		***Cognitive***	***Attitudinal***	***Behavioral***
Composition	*Modality*		(19)	
	*Centrality*			
	*Duration*			
	*Interaction*	(32, 54)	–	(61)
Source	*Type of Characters*	(73)	(70)	(12, 22, 71, 72, 80)
	*Consensus of Characters*	–	–	(22, 61)
Information	*Emphasis Frames*	(30)	(30)	(30, 86)
	*Equivalence Frames*	(80)	(93)	(80)

**Table 2:** Overview of Evidence on the Persuasive Strategies Regarding the Effects of Healthy Foods (HF) on Children.

		**Outcomes**
Composition-related Characteristics	Modality	Audiovisual HF presentations of HF are more effective in creating HF intentions in children compared to visual presentations (19)
	Centrality	No insights on the centrality of food presentations on children's response to HF presentations to date
	Duration	No insights on the duration of food presentations on children's response to HF presentations to date
	Interaction	Interactive HF presentations create more awareness/memory for these foods (32, 54); Of what people are actually doing, affect children's snack choices (61)
Source-related Characteristics	Type of Characters	Expert recommendations (74) and perceived majority preferences for a certain food in peer groups (22) can positively impact children's HF choice, while celebrity spoke persons positively affect attitudes toward HF (70) but do not impact young people's awareness for HF (73) nor HF choice (12, 71, 72)
	Consensus of Characters	Presenting a majority of characters (22) connected with HFs and transporting relevant social norms (61) positively impacts children's HF choice
Information-related Characteristics	Emphasis Frames	Positive affective cues compared to cognitive cues positively impact children's assessment of HF and their HF choices (30). Threat appeals proofed more effective than fun and action appeals to impact young people's health food consumption (86). No insights on appearance/health frames or favored outcomes on children's response to HF presentations to date
	Equivalence Frames	Gain compared to loss arguments in connection with HF presentations positively influence children's attitudes, intentions, and real consumption behavior (80, 93). There are no insights of consequences on children's response to HF presentations to date

### Discussion

The aim of this article was to provide researchers, health promoters, and program planners with a holistic “blueprint” of possibilities to integrate HFs within the media content of children, the PSPHF typology. The review of theoretical assumptions and empirical evidence also provides a valuable overview of the complexity of the effects of HF media presentations on children. Significant gaps still remain to understand the effectiveness of different message factors in connection with HFs.

We lack research looking into how composition-related factors of HFs shape cognitive, attitudinal, and behavioral outcomes in children. This is surprising because composition-related factors are the most basic characteristics of messages, and they are also comparatively easy to vary. Cognitive outcomes, prompted by composition-related factors, are particularly relevant, because they may drive attitudinal and behavioral outcomes. Some studies showed that audiovisual presentations (19) and interactive elements can contribute to some positive effects in children (32, 60). Research using eye-tracking, or heart rate measures (29), might lead to even more insights into how these factors influence the reactions of children toward HFs. To our knowledge, only two studies used eye-tracking measures to investigate the effects of HFs on children (32, 54). Other studies should follow these examples.

With regard to source-related aspects, studies with regard to HFs in audiovisual media are still inconclusive. First empirical evidence shows some positive effects with regard to peers (22), experts (74), and celebrities (70) presentations with HFs. However, not all source-related factors have been systematically studied to date. In this context, evaluative outcomes seem to be most pressing. Depending on the type of endorsers presented in connection with HFs, different underlying mechanisms might be prevalent (75). To gain better insights into how and why specific endorsers are especially effective, it seems important to conduct qualitative studies to gain a deeper understanding of these processes as well as physiological studies that highlight cue reactivity responses of children to different characters. Since especially familiar and popular endorsers seem to be effective (60), studies should first set their focus on endorsers children already know. From the current research, using a majority of endorsers seems to be especially effective or at least not harming the effects of HF presentations (22, 61).

The third pillar, information-related characteristics, can be described as the most heterogeneous one. While some presentation strategies have been found to influence the healthy eating behavior of children positively (e.g., gain framing, affective cognitive arguments) (30, 80, 93), other components have been hardly (e.g., threat appeals) (86), or not at all studied (appearance- vs. health-framing, reward presentations) (64, 89). Thus, qualitative studies are needed as a first step to gain detailed insights into how children evaluate and react when being exposed to these message factors.

#### Implications for Future Research

Our suggested typology, along with the discussion of prior research, bears a number of important theoretical and methodological implications. In fact, with the exception of interactive elements, most composition-related factors are relevant for creating awareness for HF presentations. Based on the available empirical evidence, one could argue that awareness alone is not sufficient to shape the healthy eating preferences of children. Composition-related factors may help to foster and support the effectiveness of source- and information-related characteristics. Source-related factors, by contrast, clearly directly affect cognitive, attitudinal, and behavioral outcomes. They relate to cognitive outcomes because endorsers, such as celebrities or popular characters, drive attention. However, they also directly affect associative processes (i.e., connect foods to evaluations) and stimulate heuristic decision-making, and thus impact evaluative outcomes such as liking and behaviors. Information-related characteristics matter for argument-based strategies, most likely under situations of high processing motivation. Since we lack studies to verify these assumptions, as a further step, the presented strategies should be tested with a series of empirical studies. Furthermore, content analyses could give insights about how these strategies have already been used for the presentation of UHF and HF. Besides the obvious lack of research for some characteristics with respect to some outcomes, we particularly identify four pressing research gaps.

First, further research should set its focus on healthy eating *behaviors* in children as an outcome variable. Studies suggest that attitudes and intentions (19, 20, 70, 93) can be influenced with HF presentations, while studies measuring eating behaviors are less conclusive (17, 21, 22). To counteract the rising overweight and obesity in children, influencing the behavior of the children seems to be one key aspect.

Second, we lack research comparing the effectiveness of composition-, source-, and information-related characteristics. For instance, some composition-related characteristics may be more relevant for cognitive outcomes compared to others. That is, the factors that drive the obtrusiveness of composition-related characteristics to children are far from being fully understood. Moreover, we lack studies on perceptual processes, using eye-tracking studies and designs systematically varying composition-related characteristics. By the same token, there is a dearth of studies systematically comparing the effects of source-related characteristics. Although there are some preliminary studies comparing the effects of peers, celebrities, and experts (74), we lack a deeper understanding of why some sources may be more consequential for cognitive, attitudinal, or behavioral outcomes than others.

Third, an important avenue to investigate is the interplay of different message factors. Composition-related characteristics may be important boundary conditions for the effectiveness of source- and information-related characteristics. That is, composition-related characteristics drive attention, and such attention can further support how, for instance, expert statements are processed, or messages are understood and stored in the memory of the children. Along the same lines, specific source-related characteristics may further support the persuasiveness of arguments made about healthy nutrition. However, the three characteristics may also work in opposite directions. One might argue that using the most effective persuasive strategies all in one can act as the ultimate motivator for healthy eating habits. However, this high persuasion attempt might also lead to reactance, especially if children gain the impression that they should be influenced by these presentations (97). Thus, investigating the interplay and closely connected to this level of persuasion attempt can lead to important insights.

Fourth, the effectiveness of composition-, source-, and information-related characteristics may depend on the individual susceptibility as well as contextual factors. According to the REFCAM (24) and the Promotion of HFs Model (23), factors such as the BMI or parental mediation styles play a key role. Studies showed that these components can shape how children react to HF presentations (16, 21, 22). Likewise, the developmental stage of children may greatly matter as well (30), which is far from being fully understood. Especially, information-related characteristics demand cognitive resources and skills that develop over time (42). Also, persuasion knowledge depends on the cognitive development of children and may predict how source- and information-related characteristics are processed and understood.

#### Practical Implications

For health promoters, and content creators, the PSPHF typology provides an overview of the different factors that can (and also should) be taken into account when HFs are integrated in media targeted at children. Our overview suggests that the mere presentation of HFs is not sufficient to generate desired outcomes. Since message factors can be directly influenced as compared to individual susceptibility characteristics and social context factors, our PSPHF typology gives clear hints about concrete stylistic elements, program plots, or relevant verbal or visual integrations. Our overview of the existing research helps to determine for which characteristics are backed up with the existing empirical research, and which are not. This helps practitioners to build their content decisions on a clear body of scientific evidence.

## Conclusion

Health promoters and content creators who aim to foster healthy eating habits of children face the challenge that the key factors driving HF choice, such as individual or social aspects, cannot be directly influenced. Message factors are thus the most important vehicle to influence HF behaviors in children. That is, message factors can be directly influenced and therefore used to counteract to the rising obesity and overweight in children. We have suggested the PSPHF typology to enable a systematic overview of message factors. Our typology clearly shows that several significant blind spots remain when trying to understand how messages can and should be drafted in order to foster healthy habits among children. At the same time, it demonstrates the empirical evidence, therefore informing not only researchers but also content creators. It is our hope that the PSPHF typology, as a general framework, sparks research in this area, potentially leading to substantial updates and also revisions of the typology.

## Author Contributions

AB, BN, and JM contributed to the conception of the review. AB conceptualized the first draft of the review and the typology. AB and BN drafted the initial manuscript and reviewed and revised the manuscript. JM wrote sections of the manuscript and reviewed and revised the manuscript. All authors approved the final manuscript as submitted and agreed to be accountable for all aspects of the work.

## Conflict of Interest

The research was supported by funds of the Oesterreichischen Nationalbank (Austrian Central Bank, Anniversary Fund, Project Number: 17 715). The authors declare that the research was conducted in the absence of any commercial or financial relationships that could be construed as a potential conflict of interest.

## Publisher's Note

All claims expressed in this article are solely those of the authors and do not necessarily represent those of their affiliated organizations, or those of the publisher, the editors and the reviewers. Any product that may be evaluated in this article, or claim that may be made by its manufacturer, is not guaranteed or endorsed by the publisher.
